# Giant enhancement in critical current density, up to a hundredfold, in superconducting NaFe_0.97_Co_0.03_ As single crystals under hydrostatic pressure

**DOI:** 10.1038/srep10606

**Published:** 2015-06-01

**Authors:** Babar Shabbir, Xiaolin Wang, S. R. Ghorbani, A. F. Wang, Shixue Dou, X. H. Chen

**Affiliations:** 1 Spintronic and Electronic Materials Group, Institute for Superconducting and Electronic Materials, Australian Institute for Innovative Materials, University of Wollongong, North Wollongong, NSW 2522, Australia; 2 Department of Physics, Ferdowsi University of Mashhad, Mashhad 9177948974, Iran; 3 Department of Physics, University of Science and Technology of China, Hefei, 230026, P. R. China

## Abstract

Tremendous efforts towards improvement in the critical current density “*J*_c_” of iron based superconductors (FeSCs), especially at relatively low temperatures and magnetic fields, have been made so far through different methods, resulting in real progress. *J*_c_ at high temperatures in high fields still needs to be further improved, however, in order to meet the requirements of practical applications. Here, we demonstrate a simple approach to achieve this. Hydrostatic pressure can significantly enhance *J*_c_ in NaFe_0.97_Co_0.03_As single crystals by at least tenfold at low field and more than a hundredfold at high fields. Significant enhancement in the in-field performance of NaFe_0.97_Co_0.03_As single crystal in terms of pinning force density (*F*_p_) is found at high pressures. At high fields, the *F*_p_ is over 20 and 80 times higher than under ambient pressure at12 K and 14 K, respectively, at *P* = 1 GPa. We believe that the Co-doped NaFeAs compounds are very exciting and deserve to be more intensively investigated. Finally, it is worthwhile to say that by using hydrostatic pressure, we can achieve more milestones in terms of high *J*_c_ values in tapes, wires or films of other Fe-based superconductors.

Significant advances towards high performing Fe-based superconducting materials (FeSCs) have been made so far, because the combination of reasonable value of critical temperature (*T*_c_), extremely high upper critical field (*H*_c2_) on the order of 100 T, high intrinsic pinning potential, low anisotropy (generally between 1-8), and high irreversible field (*H*_irr_) makes this class of superconductors particularly attractive for large current and high field applications, where the critical current density (*J*_c_) is a major limiting factor^1^,^2^,^3^,^4^,^5^,^6^,^7^,^8^,^9^,^10^,^11^,^12^,^13^,^14^. Therefore, improvement in *J*_c_ by using various methods has also been one of the most important topics in the superconductivity research field. Texturing procedures, ion implantation/irradiation, and chemical doping are the most common approaches to enhancing *J*_c_ in different superconductors. Although *J*_c_ values can be improved by these methods, the major drawbacks are that *J*_c_ decays rapidly in high fields, especially at high temperatures. Therefore, the *J*_c_ values of the Fe-based superconductors at high fields and temperatures need to be improved. Furthermore, *T*_c_ and low field *J*_c_ deteriorate significantly for various types of superconductors under these approaches, which make them rather impractical for application. The reported enhancement in *J*_c_ values at high fields and temperatures is still not more than one order of magnitude.^5^,^15^,^16^,^17^,^18^,^19^,^20^,^21^,^22^. Generally, the requirements for enhancing *J*_c_ in superconductors include *T*_c_ enhancement, which can increase the effective superconducting volume, and the formation of more effective point pinning centres (related to the pinning mechanism).

Hydrostatic pressure has many significant impacts on Fe-based superconductors. For instance, pressure can raise the onset *T*_c_ up to 50 K at 1.5 Gpa for LaFeAsO_δ_^23^. The application of pressure on BaFe_1.92_Co_0.08_As_2_ results in a very strong enhancement of *T*_c_ from 11 to 21 K at 2.5 Gpa^24^. For Co-doped NaFeAs, enhancement in *T*_c_ is more than 14 K at 2.5 GPa due to optimization of the structural parameters of the FeAs layers^25^. The *T*_c_ of FeSe is enhanced up to 37 K at 7 Gpa^26^. Furthermore, pressure can induce reduction in anisotropy, more effective point pinning centres, and enhancement in *T*_c_. We already anticipated in our previous case study that the most significant approach to enhancing Jc, particularly at high fields and temperatures, without degradation of Tc, is the use of hydrostatic pressure^27^.

Most recent research regarding *J*_c_ enhancement and pinning mechanisms is mainly focused on the 1111 system (RFeAsO, where R is a rare earth element), the 122 system (BaFe_2_As_2_, Ba_0.5_K_0.5_Fe_2_As_2_) and the iron chalcogenide 11 system. Only one report has revealed the nature of the pinning mechanism in LiFeAs (111 type FeSCs) so far, despite its simple structure and reasonable *T*_c_ value as compared to the 1111 and 122 types^28^. NaFeAs (*T*_c_ ≈ 10 K) experiences three successive phase transitions around 52, 41 and 23 K, which can be related to structural, magnetic, and superconducting transitions, respectively^29^,^30^,^31^. Bulk superconductivity in NaFeAs with *T*_c_ of ~ 20 K can be achieved by the substitution of Co on Fe sites, which can suppress both magnetism and structural distortion^25^,^32^. The *T*_c_ of NaFe_0.97_Co_0.03_As single crystal is more sensitive to hydrostatic pressure as compared to other 11 and 111 Fe-based superconductors, and it has a large positive pressure coefficient^25^. In addition, *J*_c_ values for Co-doped NaFeAs compounds have not been reported so far. Therefore, it is very interesting to see if the hydrostatic pressure can significantly improve the flux pinning for such compounds. High-quality NaFe_0.97_Co_0.03_As single crystals were grown by the conventional high temperature solution growth method using the NaAs self-flux technique^30^. In this communication, we report that hydrostatic pressure can enhance the *J*_c_ by more than 100 times at high fields at 12 K and 14 K in NaFe_0.97_Co_0.03_As single crystal. This is a giant enhancement of *J*_c_ and a record high to the best of our knowledge. The *H*_irr_ is improved by roughly 6 times at 14 K under *P* = 1 GPa.

The temperature dependence of the magnetic moment for zero-field-cooled (ZFC) and field-cooled (FC) curves at different pressures are shown in Fig. 1. The *T*_c_ increases with pressure, from 17.95 K for *P* = 0 GPa to 24.33 K for *P* = 1 GPa, with a huge pressure coefficient, i.e. *dT*_c_/*dP ~* 6.36 K/GPa, which is nearly same to what we have already reported for NaFe_0.97_Co_0.03_As single crystal i.e. *dT*_c_/*dP* = 7.06 K·GPa^−1^^25^. Interestingly, this pressure coefficient is more than two times greater than that of FeSe (3.2 K·GPa^−1^)^33^. The pressure-induced enhancement of *T*_c_ in NaFe_0.97_Co_0.03_As can be associated with the optimization of the structural parameters of the FeAs layers, including the As–Fe–As bond angle and anion height^25^,^34^.

The field dependence of *J*_c_ at different temperatures obtained from the *M*-*H* curves by using Bean’s model, at *P* = 0 GPa, *P* = 0.45 GPa and *P* = 1 GPa are shown in Fig. 2. Remarkably, *J*_c_ is increased significantly at both low and high fields, especially with enhancement of more than 10 times and up to more than 100 times for low and high fields at both 12 and 14 K, respectively. The significant positive effect of hydrostatic pressure on the *J*_c_ at high fields and temperatures is further reflected in Fig. 3, which shows the *J*_c_ enhancement ratio (i.e.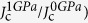 at 12 and 14 K over a wide range of fields. We have taken the *J*_c_ value at *P* = 0 GPa as a reference. The *J*_c_ ratio values at both temperatures show significant improvements at low and high fields. Although this result also suggests that hydrostatic pressure is more effective at high fields and temperatures, it is worth mentioning that *J*_c_ values are well improved at zero field at a significant rate, i.e. *d*(ln*J*_c_)/*dP* = 1.6 and 2.9 GPa^−1^ at 12 and 14 K, respectively, as can be seen from the inset of Fig. 3. The *d*(ln*J*_c_)/*dP* values that have been found are more significant than for yttrium barium copper oxide(YBCO)^35^.

We also found that *H*_irr_ of NaFe_0.97_Co_0.03_As is significantly increased by pressure. As shown in Fig. 4, the *H*_irr_ values improve gradually with pressure, and the H_irr_ value at 14 K is increased from 2.6 T at P = 0 GPa up to 8.67 T at P = 0.45 GPa and roughly more than 13 T at P = 1 GPa (by nearly six times).

In Fig. 5, we show the temperature dependence of *J*_c_ at 0 and 12 T under different pressures. It follows power law [*J*_c_ ∝ (1-*T*/*T*_c_)^β^] behaviour at different pressures. According to the Ginzburg-Landau theory, the exponent *β* is used to identify different vortex pinning mechanisms at specified fields. It was found that *β* = 1 refers to non-interacting vortices and *β* > 1.5 corresponds to the core pinning mechanism^26^. The exponent *β* (i.e. slope of the fitting line) is found to be 1.79 and 1.85 for zero field, and 2.73 and 4.28 at 12 T, at 0 and 1 GPa, respectively, which shows strong *J*_c_ dependence on pressure. The low values of *β* at *P* = 1 GPa indicate that the *J*_c_ decays rather slowly in comparison to its values at *P* = 0 GPa. In addition, the differences between *P* = 0 GPa and *P* = 1 GPa scaling show a real pressure effect, the factor is roughly 2, which corresponds nicely to the low-T data in inset Fig. 3.

For polycrystalline bulks, high pressure can modify grain boundaries through reduction of the tunnelling barrier width and the tunnelling barrier height. The Wentzel-Kramers-Brillouin (WKB) approximation applied to a potential barrier gives the following simple expression^36^,^37^,^38^:
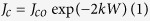


Where *W* is the barrier width, *k* = (2 *mL*)^1/2^/ħ is the decay constant, which depends on the barrier height *L*, ħ; is the Planck constant, and *J*_c0_ is the critical current density at 0 K and 0T. The relative pressure dependence of *J*_c_ can be obtained from Eq. (1) as^39^:
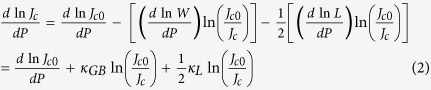


Where the compressibility in the width and height of the grain boundary are defined by 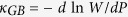 and , respectively. For single crystals, we assume to a first approximation that *κ*_GB_ and *κ*_L_ are roughly comparable, respectively,to the average linear compressibility values *κ*_a_ = –*d*ln*a*/*dP* (*κ*_a_≈–0.029 GPa^−1^) and *κ*_c_ = –*d*ln*c*/*dP* (*κ*_c_≈–0.065 GPa^−1^) of NaFe_0.97_Co_0.03_As crystal in the FeAs plane, where *a* and *c* are the in-plane and out-of-plane lattice parameters, respectively^26^. Therefore, we can write Eq. (2) as
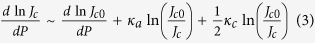


By using *J*_c_≅1.3 × 10^3^  A/cm^2^ at 14 K and *J*_c0_ ~ 10^5^ A/cm^2^, we find that (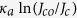) ≈ 0.12 GPa^−1^ and (1/2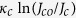) ≈ 0.14 GPa^−1^, so that both of them together only contribute less than 10% to the already mentioned experimental value *d*ln*J*_c_/*dP* = 2.09 GPa^−1^ inset of Fig. 2b). This result suggests that the origin of the significant increase in *J*_c_(*T*) under pressure does not arise from the reduction of volume but mainly due to the pressure induced pinning centre phenomenon.

To gain further insight into the pressure effect on the pinning mechanism in NaFe_0.97_Co_0.03_As, the experimental results have been analysed by using collective pinning theory. There are two predominant mechanisms of core pinning, i.e. 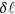 pinning, which comes from spatial variation in the charge carrier mean free path,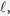 and *δT*_c_ pinning due to randomly distributed spatial variation in *T*_c_. According to the theoretical approach proposed by Griessen *et al*.^40^, 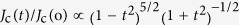 in case of 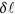 pinning, whereas 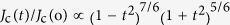 corresponds to *δT*_c_ pinning, where 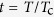 Fig. 6 (Top Panel) shows a comparison between the experimental *J*_c_ values and the theoretically expected variation within the 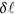 and *δT*_c_ pinning mechanisms at 0.1 T and 0 T (the so-called remanent state shown by the solid symbols). The *J*_c_(*t*) values have been obtained from the *J*_c_(*B*) curves at various temperatures. It is found that the experimental data at *P* = 0 and *P* = 1 GPa are in good agreement with theoretical 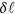 pinning. It is more likely that pinning in NaFe_0.97_Co_0.03_As originates from spatial variation of the mean free path “
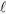
”. We observed similar results in BaFe_1.9_Ni_0.1_As_2_ and SiCl_4_ doped MgB_2_ at low fields. In addition, 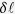 pinning has also been reported in FeTe_0.7_Se_0.3_ crystal^41^,^42^,^43^. In order to understand the nature of the pinning mechanisms in more detail, it is useful to study the variation of the vortex pinning force density, with the field. The normalized pinning force density (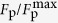) as a function of reduced field (*H*/*H*_irr_) at *P* = 0 GPa and *P* = 0.45 GPa at 14 K is plotted in lower panel of Fig. 6. *H*_irr_ is estimated by using the criterion of Jc ~100A/cm^2^. We can use the Dew-Hughes formula, i.e. *F*_p_ ∝ *h*^*m*^(1 − *h*^*n*^) to fit our experimental data, where *m* and *n* are fitting parameters to describe the nature of the pinning mechanism. We found that *m* = 1.15 and *n* = 2 at 0 GPa, and *m* = 1.1 and *n* = 2.1 at 0.45 GPa. According to the Dew-Hughes model, in the case of 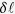 pinning for a system dominated just by point pinning, the values of the fitting parameters are *m* = 1 and *n* = 2, with the 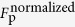
maxima occurring at *h*_max_ = 0.33, while *h*_max_ occurs at 0.20 for surface/grain boundary pinning with *m* = 0.5 and *n* = 2. In case of *δT*_c_ pinning, *h*_max_ shifts to higher values, and the fitting parameters change accordingly. Further details can be found elsewhere^44^. The values of *m* and *n* that were found in the present study are almost the same at 0 GPa and 0.45 GPa, so normal core point pinning is dominant in our material.

Pressure can enhance the pinning force strength by a significant amount in NaFe_0.97_Co_0.03_As single crystal. The pinning force density as a function of field at 12 K and 14 K is plotted in Fig. 7. At high field and pressures, the *F*_p_ is found to be over 20 and 80 times higher than at 0 GPa at 12 and 14 K, respectively. Furthermore, pressure induces more point pinning centres at 12 K and 14 K, especially at *P* = 1 GPa, as can be seen in the inset of Fig. 7. The number density of randomly distributed effective pinning centres (*N*_p_) can be calculated from the following relation:
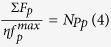


Where Σ*F*_P_ is the aggregated pinning force density, 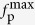 is the maximum normalized elementary pinning force (*f*_p_), and *η* is an efficiency factor. The *η* value is 1 in the case of a plastic lattice, and the *η* value is otherwise 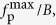 where *B* is the bulk modulus of the material^45^. We can assume that *η* = 1, as pressure can shrink lattice parameters. The inset of Fig. 7 shows the *N*_p_ versus temperature plot at *P* = 0 and *P* = 1 GPa. The *N*_p_ values are found to be much greater at 14 K at *P* = 1 GPa as compared to *N*_p_ at *P* = 0 GPa (nearly six times as great). It is well known that hydrostatic pressure induces pinning centres which, in turn, leads to huge values of *J*_c_ and increase in *N*_p_ at P = 1 Gpa is a direct evidence of that^27^,^46^,^47^,^48^. This is further verified in Fig. 8, which shows the plot of 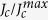 versus reduced field (i.e. H/H_*irr*_) at *P* = 0 GPa and *P* = 1 GPa for 14 K and *P* = 0 GPa & *P* = 0.45 GPa for 12 K. Obviously, the hump or secondary peak effect observed at high pressures suggests that the *J*_c_ enhancement is due to induced pinning centres.

Additionally, we found a pronounced reduction in the superconducting anisotropy at high temperatures, by almost 63% at *P* = 1 GPa. The pressure dependence of the *T*_c_, volume (*V*) and anisotropy (*γ*) are interconnected through a relation^49^:.
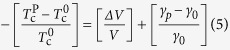


At Δ*P* = 1 GPa, Δ*V*(T_c_)/*V*(T_c_) is estimated to be –0.02, as Δ*V*/*V* = –Δ*P*/B, where *B* is the bulk modulus. We can use the bulk modulus (B≈52.3 GPa) of a similar superconductor, i.e. Na_1-*x*_FeAs^34^. The value of *γ* at *P* = 1 GPa is found to be 63% less than its value at *P* = 0 GPa.

Hydrostatic pressure can significantly enhance *J*_c_ by up to 10^2^ times in NaFe_0.97_Co_0.03_As single crystals, which is a record high enhancement. The most significant enhancement in in-field performance of NaFe_0.97_Co_0.03_As in terms of pinning force density (*F*_p_) is found at *P* = 1 GPa in particular, where the *F*_p_ at high fields is over 20 and 80 times higher at 12 and 14 K, respectively, than at 0 GPa. The hydrostatic pressure induces more effective point pinning centres and *N*_p_ at 1 GPa is almost two times higher at 12 K and over six times higher at 14 K compared to the value at 0 GPa. Moreover, a hump or secondary peak effect is found from the plot of the normalized *J*_c_ as a function of reduced field. Therefore, this giant enhancement in *J*_c_ values for NaFe_0.97_Co_0.03_As exists because of more pinning centres induced by pressure and the increase in pinning strength as well. The present study indicates that the supercurrent carrying ability in the Fe111 can be further and significantly increased by the proposed hydrostatic pressure technique. Our results were achived in single crystal samples, which means that the enhancement is intrinsic, and more significant than other reported approaches. It gives us high expections that the tapes or wires made by the same compounds should carry higher supercurrents using the hydrostatic pressure than those at ambient pressure.

## Experimental

High-quality single crystals of NaFe_0.97_Co_0.03_As have been grown by use of the NaAs flux method. NaAs was obtained by reacting the mixture of the elemental Na and As in anevacuated quartz tube at 200^°^C for 10 h. Then NaAs, Fe and Co powders were carefully weighed according to the ratio of NaAs:Fe:Co = 4:0.972:0.028, and thoroughly ground. The mixtures were put into alumina crucibles and then sealed in iron crucibles under 1.5 atm of highly pure argon gas. The sealed crucibles were heated to 950^°^C at a rate of 60 ^°^C/h in the tube furnace filled with the inert atmosphere and kept at 950 ^°^C for 10 h and then cooled slowly to 600 ^°^C at 3 ^°^C/h to grow single crystals.

The temperature dependence of the magnetic moments and the M-H loops at different temperatures and pressures were performed on Quantum Design Physical Property Measurement System (QD PPMS 14 T) by using Vibrating Sample Magnetometer (VSM). We have used HMD High Pressure cell and Daphne 7373 oil as a pressure transmitting medium to apply hydrostatic pressure on a sample. The critical current density was calculated by using the Bean approximation.

## Additional Information

**How to cite this article**: Shabbir, B. *et al*. Giant enhancement in critical current density, up to a hundredfold, in superconducting NaFe_0.97_Co_0.03_ As single crystals under hydrostatic pressure. *Sci. Rep*. **5**, 10606; doi: 10.1038/srep10606 (2015).

## Figures and Tables

**Figure 1:**
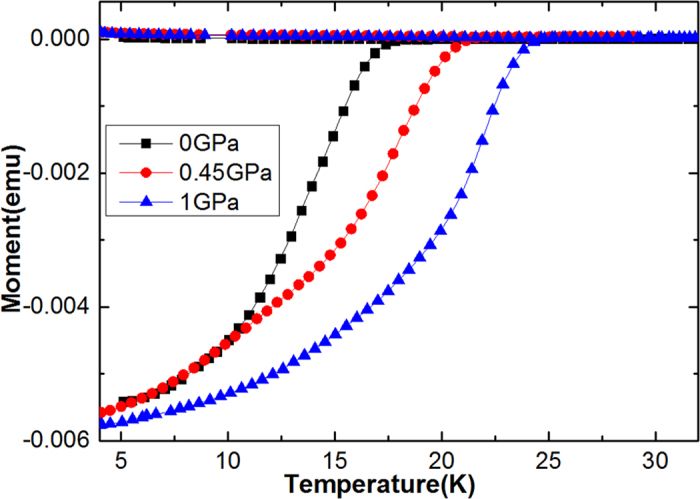
Temperature dependence of magnetic moment at different applied pressures in both ZFC and FC runs for NaFe_0.97_Co_0.03_As.

**Figure 2:**
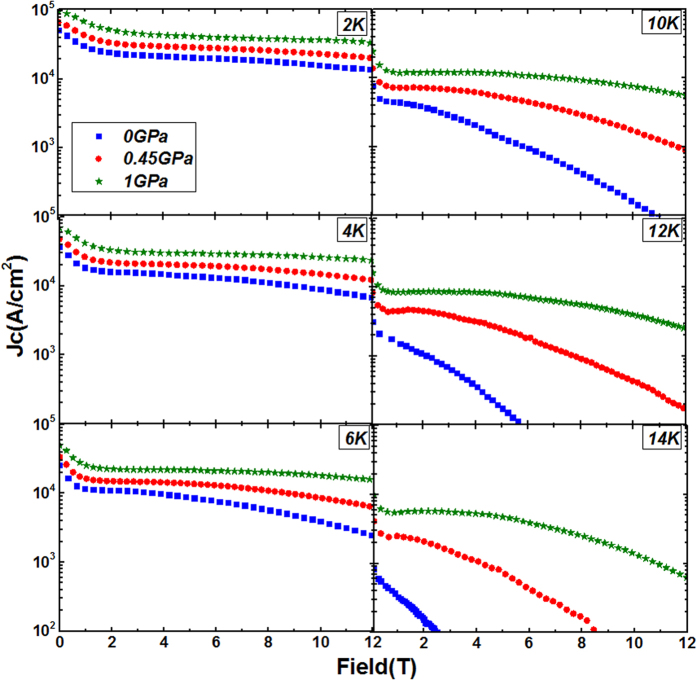
Field dependence of *J*_c_ at different pressures (0, 0.45 and 1 GPa) at different temperatures.

**Figure 3:**
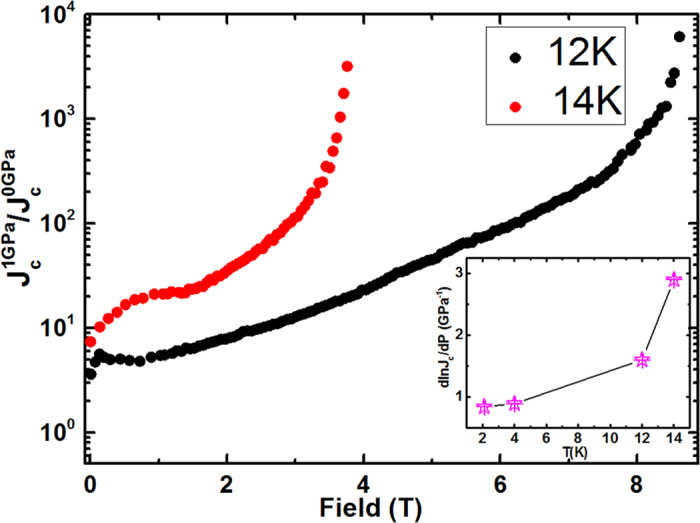
Plot of 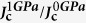 versus field at 12 K and 14 K. There is a giant enhancement in *J*_c_ values at *P* = 1 GPa. The inset shows the plot of *d*(ln*J*_c_)/*dP* versus temperature, which demonstrates enhancement in ln*J*_c_ at a rate of nearly 3 GPa^−1^ at 14 K at zero magnetic field.

**Figure 4:**
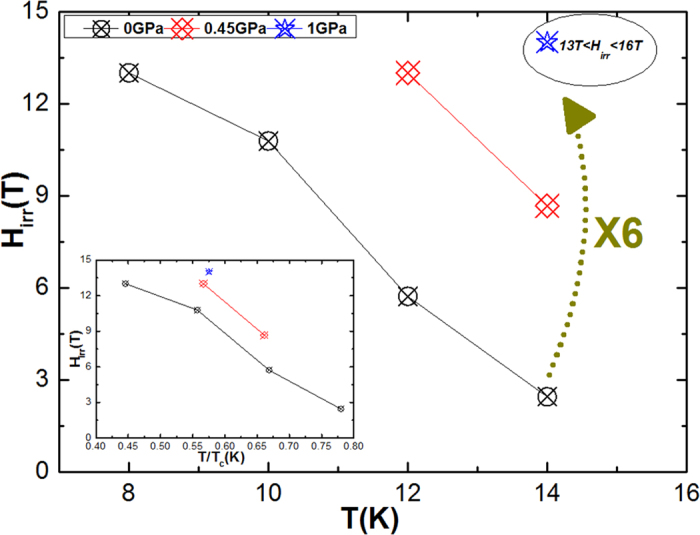
Plot of *H*_irr_ versus temperature at different pressures. Inset shows *H*_irr_ as a function of reduced temperature.

**Figure 5:**
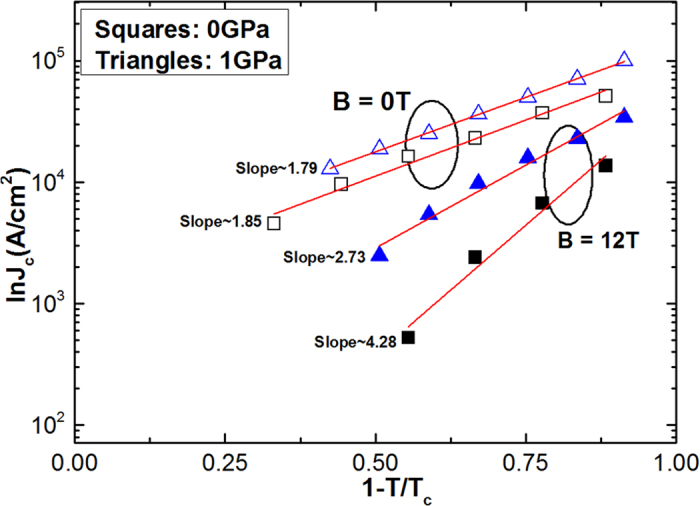
Logarithmic plot of critical current density as a function of reduced temperature at different pressures and magnetic fields.

**Figure 6:**
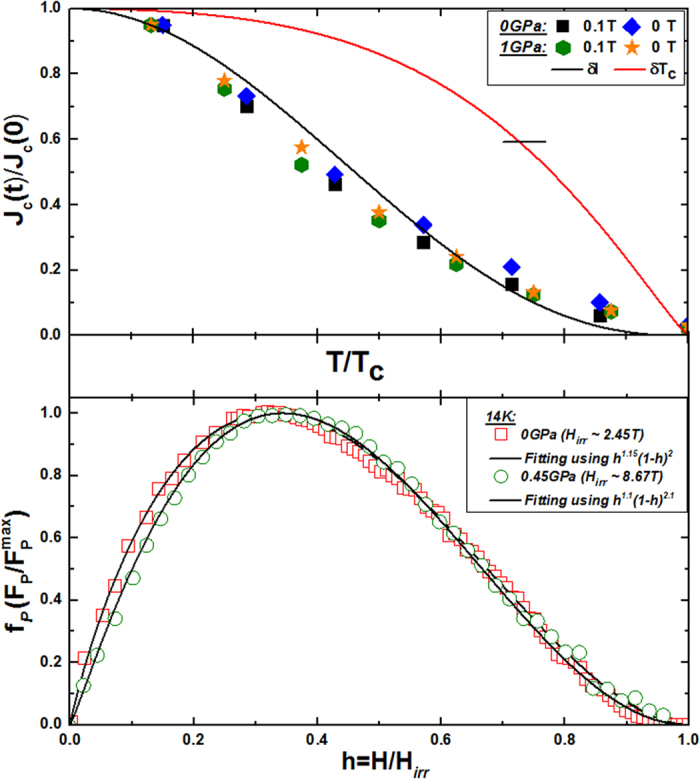
Top panel shows normalized temperature dependence (*t* = *T*/*T*_c_) of normalized measured *J*_c_ at 0.1 T and 0 T, in good agreement with ***δ**l* pinning. Lower panel shows plots of *F*_p_ vs *H*/*H*_irr_ at *P* = 0 GPa and *P* = 0.45 GPa at 14 K. The experimental data is fitted through the Dew-Hughes model, and the parameters are given in inset.

**Figure 7:**
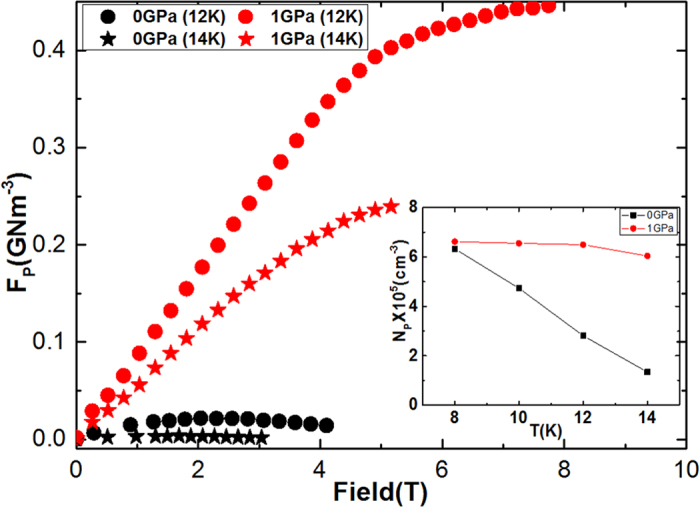
Pinning force density (*F*_p_) as a function of field at *P* = 0 GPa and *P* = 1 GPa at 12 K and 14 K. The inset shows the temperature dependence of the pinning centre number density at the different pressures.

**Figure 8:**
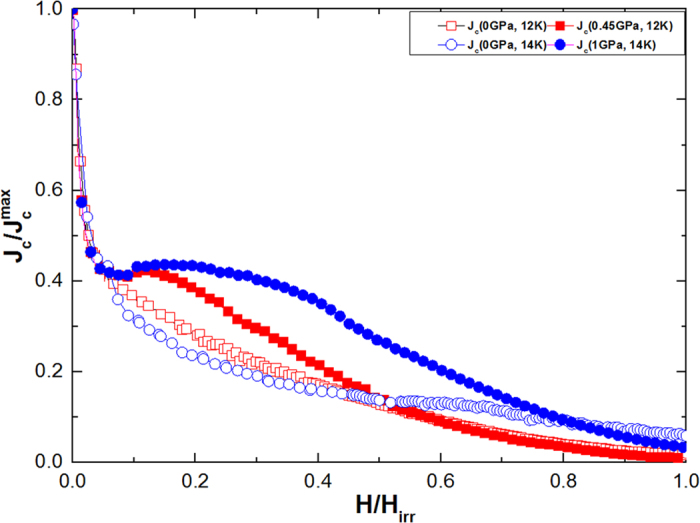
Reduced field dependence of the normalized *J*_c_ at 14 K for different pressures. The inset shows the same plot for 12 K at *P* = 0 GPa and *P* = 0.45 GPa.
